# Meningococcemia Masquerading as a Nonspecific Flu-Like Syndrome

**DOI:** 10.1155/2018/2097824

**Published:** 2018-11-05

**Authors:** Paula J. Watts, Natasha Fazel, Dmitriy Scherbak

**Affiliations:** ^1^Sky Ridge Medical Center, 10099 RidgeGate Parkway, Lone Tree, CO 80124, USA; ^2^Rocky Vista University, 8401 South Chambers Rd, Parker, CO 80134, USA; ^3^Critical Care and Pulmonary Consultants, Denver, CO, 5200 DTC Parkway Suite 400, Greenwood Village, CO 80111, USA

## Abstract

*Neisseria meningitidis* is a cause of bacterial meningitis and meningococcemia worldwide. Rarely, it causes invasive disease with significant lifelong sequela if survived. Early clinical recognition is key as meningococcemia is an easily treatable disease, yet mortality is 50% if it is left untreated. In this case review, we present a classic case of meningococcemia, with an atypical presentation.

## 1. Introduction

*Neisseria meningitidis* is a gram-negative aerobic diplococcus, a cause of bacterial meningitis and meningococcemia worldwide [1]. It is a common bacterial commensal of the human nasopharynx with no animal reservoir [2, 3]. Its incidence differs drastically around the world, based on the serogroup virulence and host immune system [4]. In this article, we aim to present a case of meningococcemia presenting as flu-like symptoms complicated by shock and purpura fulminans.

## 2. Case Presentation

A 61-year-old otherwise healthy Caucasian male presented to our institution with nonspecific, flu-like symptoms. The patient had been traveling with his wife to Austria, Switzerland, and Germany the month prior to presentation. They had taken a riverboat, had been in very close proximity to other people on the cruise, and there were multiple individuals who reportedly experienced similar symptoms. Upon return to the U.S., the patient's symptoms had initially improved. However, they traveled to Creede, Colorado, where his symptoms then progressed approximately 4–16 days after potential exposure.

Initially in the emergency department, the patient complained of malaise, dyspnea, chills, mild intermittent headache, and a fever. He quickly decompensated with high fevers, tachycardia, leukopenia, and a lactic acid of 7.69. Infectious etiology was considered likely. The patient was placed on broad-spectrum antibiotics; vancomycin, piperacillin/tazobactam, and levofloxacin, and given aggressive fluid resuscitation. He was admitted to the intensive care unit and within hours deteriorated further, requiring intubation, three vasopressors, and continuous renal replacement therapy. Within twelve hours, blood cultures returned positive for gram-negative diplococci, later identified as* N. meningitidis* W135 serotype. Over the subsequent hospital days, the patient went into disseminated intravascular coagulopathy (DIC) and progressed to purpura fulminans (PF). His clinical status did improve over the coming days and would eventually require bilateral transmetatarsal and digit amputations, as well as allograft over these areas.

## 3. Discussion

*N. meningitidis* occurs more often as epidemics with its largest burden found in sub-Saharan Africa and less so in developed countries [4]. It is seen in children and, more commonly, in young adults worldwide. The incubation period is between 2 and 14 days, commonly showing clinical signs by day 4 [1, 2]. There are 13 identified serotypes of* N. meningitidis*; 6 are known to cause epidemics and fatal disease: A, B, C, W135, X, and Y [1–3]. Serotypes are based on different capsular polysaccharides. W135 is typically endemic to the African meningitis belt [1, 3]. Roughly 10–20% of the population are asymptomatic carriers of* N. meningitidis *[2]. Exposure to a hypervirulent strain is responsible for pathogenicity [5].

Clinical manifestation is variable. Meningitis alone from* N. meningitidis* is the most common clinical manifestation. However, mild meningococcemia, shock with meningitis, and, rarely, shock without meningitis are other presentations. Severity is directly associated with bacterial load [5].* N. meningitidis* is one of the most highly destructive etiologies of septic shock due to its ability to cause PF and multiorgan failure.

**Figure 1:**
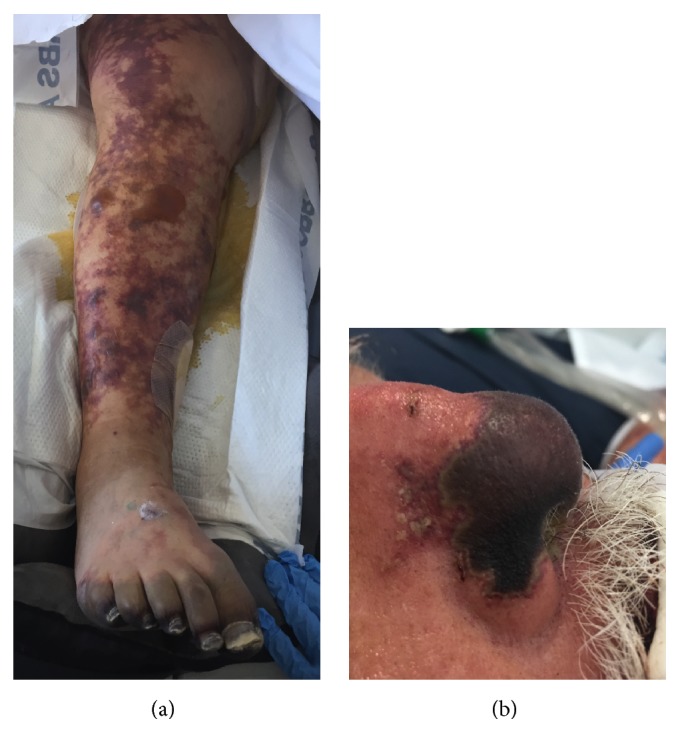
(a) Hospital day 9. Well-demarcated, large ecchymotic rash with multiple large bullae to the right lower extremity and hemorrhagic necrosis to the distal digits and foot. (b) Hospital day 9. Necrosis involving the nasal supratip, tip, septum, and ala.

*N. meningitidis* spreads by droplets or direct contact. Its virulence is attributed to its ability to evade the innate immune system via its cell wall attributes and ability to uptake L-glutamate protecting it from phagocytic cells [5]. It alters the surface of the endothelium when it adheres vigorously to cells, multiplies, and forms microcolonies. In doing so, there is loss of capillary integrity, thrombosis, and hemorrhage, eventually causing PF. PF is a rapidly progressive fatal condition, which presents as the sudden appearance of large ecchymotic areas and progresses to acral skin necrosis (Figure 1). It involves intravascular thrombosis, cutaneous infarction, and circulatory collapse.

Meningococcal risk factors include close contacts or crowding such as among college students and military recruits living in close quarters, poor socioeconomic status, immunocompromised conditions such as terminal complement pathway deficiency, human immunodeficiency virus infection, acquired complement deficiency, and splenic dysfunction. [1, 4]. One's risk is increased by other cofactors, which include smoking, coinfections with influenza, mycoplasma, or other upper respiratory tract infections [4]. In our patient case, close crowding was potentially his only risk factor while on a cruise. It is unlikely that he has an inherited or acquired complement deficiency based on his history, as he had been otherwise healthy, without recurrent infections, liver or kidney dysfunction, or autoimmune disorders.

If meningococcemia is suspected, a sample should be obtained immediately, either from a blood inoculate, cerebral spinal fluid, synovial fluid, pericardial fluid, or via skin biopsy [1]. This will confirm and facilitate rapid administration of the appropriate treatment. Initiating antibiotics immediately, ideally after a lumbar puncture and cultures have been obtained, is crucial to the patient's survival. Diagnostic procedures should never delay initiation of antibiotics. Penicillin G, ampicillin, 3^rd^-generation cephalosporins, and chloramphenicol are acceptable choices [1, 2]. However, due to resistance, chloramphenicol is only used as empiric therapy in endemic areas [1]. The Center for Disease Control reports a mortality rate of 8–15%, even with early diagnosis and treatment, and up to 50% if left untreated [2]. A high awareness of this disease is imperative regardless of age since clinical presentation is not always straightforward, as demonstrated in this case report.
